# Identification of Plasmatic Biomarkers of *Foie Gras* Qualities in Duck by Metabolomics

**DOI:** 10.3389/fphys.2021.628264

**Published:** 2021-02-12

**Authors:** Zohre Mozduri, Nathalie Marty-Gasset, Bara Lo, Ali Akbar Masoudi, Mireille Morisson, Cécile Canlet, Julien Arroyo, Agnès Bonnet, Cécile M. D. Bonnefont

**Affiliations:** ^1^GenPhySE, Université de Toulouse, INRAE, ENVT, Castanet Tolosan, France; ^2^Department of Animal Science, Faculty of Agriculture, Tarbiat Modares University, Tehran, Iran; ^3^Toxalim, Université de Toulouse, INRA, ENVT, INP-Purpan, UPS, Toulouse, France; ^4^Axiom Platform, MetaToul-MetaboHUB, National Infrastructure for Metabolomics and Fluxomics, Toulouse, France; ^5^ASSELDOR, Station d’expérimentation appliquée et de démonstration sur l’oie et le canard, La Tour de Glane, Coulaures, France

**Keywords:** liver, *foie gras*, quality, biomarker, plasma, metabolomics

## Abstract

The *foie gras* is an emblematic product of French gastronomy composed of waterfowl fatty liver. The organoleptic qualities of this product depend on the liver characteristics such as liver weight (LW) and technological yield (TY) at cooking. One of the main issues for producers is to classify the *foie gras* with high or low technological quality before cooking them. Thus the study aims at identifying biomarkers of these characteristics with non-invasive biomarkers in duck. ^1^H-NMR (nuclear magnetic resonance of the proton) analyses were performed on plasma of male mule ducks at different time points during the overfeeding period to obtain a large range of liver characteristics so as to identify plasmatic biomarkers of *foie gras*. We used two methods, one based on bucket data from the ^1^H-NMR spectra and another one based on the fingerprints of several metabolites. PLS analyses and Linear models were performed to identify biomarkers. We identified 18 biomarkers of liver weight and 15 biomarkers of technological yield. As these two quality parameters were strongly correlated (−0.82), 13 biomarkers were common. The lactate was the most important biomarker, the other were mainly amino acids. Contrary to the amino acids, the lactate increased with the liver weight and decreased with the technological yield. We also identified 5 biomarkers specific to LW (3 carbohydrates: glucuronic acid, mannose, sorbitol and 2 amino acids: glutamic acid and methionine) that were negatively correlated to liver weight. It was of main interest to identify 2 biomarkers specific to the technological yield. Contrary to the isovaleric acid, the valine was negatively correlated to the technological yield.

## Introduction

The *foie gras* of duck is a traditional product in France. It corresponds to a liver weighing more than 300 g (JORF, 1993) that is composed of 50 to 60% of lipids. During the cooking process, the lipids can melt resulting in a low cooking technological yield (TY). In addition to deteriorating the sensory qualities of the products, TY also directly influences the economic valuation of the *foie gras*. TY is controlled by the French legislation as it must exceed 70% in products labeled *Foie gras* (JORF, 1993).

The manufacturers classify the livers in function of their potential cooking TY using the liver weight (LW) and/or the liver texture. They can adapt their cooking protocol from pasteurization to sterilization to avoid the melting process and to improve TY. However the prediction of the potential cooking TY is not satisfying. As a result, a part of the cooked *foie gras* products are downgraded into products with a lower added value. One of the major challenges for manufacturers is therefore to predict TY of *foie gras* and to choose the best food processing. In addition, TY has a −0.80 genetic correlation with LW (Marie-Etancelin et al., 2011) and a −0.83 phenotypic correlation with LW (Bonnefont et al., 2019). But few authors tried to predict it before animal slaughtering. To predict phenotypes by identifying biomarkers, the plasma in an interesting fluid because plasma sampling is minimally invasive. Hermier et al. (1999) already analyzed some phospholipids of plasmatic very low density lipoprotein (VLDL) and high density lipoprotein (HDL) in control and overfed Landes goose. They showed a stronger quantity of phosphatidylcholine in VLDL and a stronger quantity of phosphatidylethanolamine in HDL in overfed goose (Hermier et al., 1999). Furthermore Tavernier et al. (2017) measured the triglycerides (TG) and non-esterified-fatty-acid (NEFA) concentrations in plasma of mule ducks during the overfeeding period. They showed a non-linear increase of TG and NEFA in parallel with LW. Recently, Pioche et al. (2019) measured the total cholesterol, the TG and the glucose in plasma of mule ducks during the overfeeding period. They highlighted a correlation between the total cholesterol and LW (0.88), between TG and LW (0.57) and between the glucose and LW (0.27) (Pioche et al., 2019). Thus these plasmatic molecules could be potential biomarkers of TY. The current study aims at identifying new biomarkers of foie gras LW and TY in mule duck.

The concentration of these metabolites directly reflects the biochemical activity and state of cells and tissues. All these metabolites can be potential biomarkers of TY that can be identify by untargeted metabolomics. Therefore, metabolomics has been strongly used to identify biomarkers (Quinones and Kaddurah-Daouk, 2009).

In the current study, plasmatic biomarkers of LW and TY were investigated by metabolomics approach using proton nuclear magnetic resonance (^1^H-NMR). The identification of LW and TY biomarkers could be used for both grading the liver of the mule ducks and increasing the added value of foie gras and improving knowledge in human hepatic steatosis.

## Materials and Methods

### Animal Experimental Design and Characteristics

**FIGURE 1:**
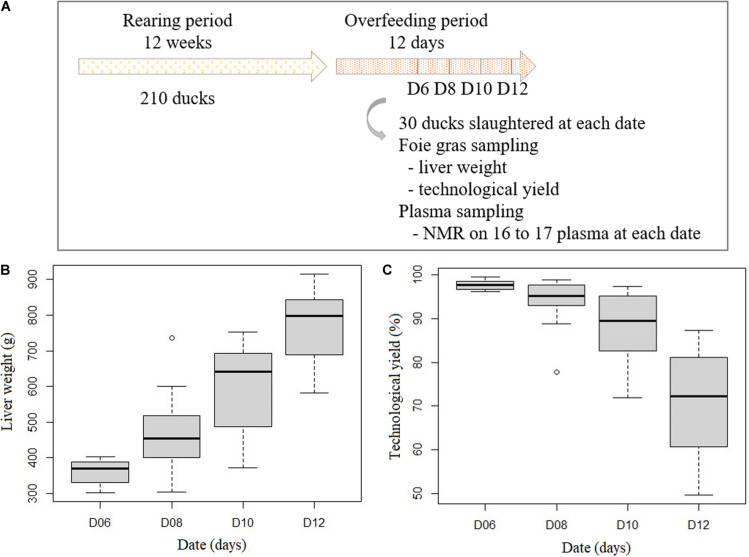
Description of the duck samples. Experimental design **(A)** Evolution of liver weight **(B)** and technological yield **(C)** during the overfeeding period (n = 16 or 17 at each time point).

The experimental protocol is clearly described in the previous paper (Bonnefont et al., 2019). Briefly, 120 male mule ducks (female *Cairina moschata* x male *Anas platyrhynchos)* were reared collectively until 12 weeks. They were overfed twice a day during 12 days with two different programs. But the overfeeding programs did not impact duck performances (Bonnefont et al., 2019) and were not taken into account in this current study. A total of 30 ducks were slaughtered at day 6, day 8, day 10 and day 12, 11 h after their last meal (Figure 1A). After 6 days of overfeeding the liver weight is expected to be superior to 300 g that corresponds to French definition of foie gras. After slaughtering the ducks, the liver was weighed. TY was determined after *foie gras* cooking as described in Rémignon et al. (2018), as following:

TY (%) = [crude weight of liver in g – (cooked weight of liver in g - visible melted lipids in g)] × 100/[crude weight of liver in g]. At each time point, 16 or 17 ducks were selected among the 30 ducks for plasma analyses (*n* = 65 in total; Figure 1A). The mean and the variability of LW and TY in the group of 30 birds and in the subgroup were equivalent. LW and TY during the overfeeding period are presented in the Figures 1B,C.

### Plasma Sampling and ^1^H-NMR Analysis

1 h before slaughtering blood was sampled from the venous occipital sinus in heparin-lithium tubes. The tubes were centrifuged at 3,400 *g* at 4°C during 10 min. The plasmas were collected and stored at −80°C for further analyses.

All plasma samples were extracted according to the procedure previously described to precipitate proteins and to avoid degraded NMR signal due to high lipids levels in fatty liver samples (Nagana Gowda et al., 2015). Briefly, 300 μL of plasma and 600 μL of methanol (MeOH) were mixed and incubated at −20°C for 20 min. After centrifugation (30 min at 11,000 *g* at 4°C), 800 μL of supernatant was isolated and evaporated using a vacuum concentrator (Concentrator plus, Eppenforf, Germany). Then, dried plasma extracts were diluted in 700 μL of NMR deuterium oxide (D_2_O) phosphate buffer (pH 7), containing sodium trimethylsilyl propionate (TSP) as an internal standard. The samples were then centrifuged at 4,600 *g* during 15 min at 4°C and 600 μl of supernatant were transferred into 5 mm NMR tubes. ^1^H-NMR analyses were performed on a Bruker Avance III HD spectrometer (Bruker Biospin, Rheinstetten, Germany) operating at a proton frequency of 600.13 MHz with an inverse detection 5-mm 1H-13C-15N cryoprobe.

^1^H-NMR spectra were acquired at 300 K with the nuclear Overhauser effect spectroscopy (NOESY) pulse sequence. A total of 128 transients were collected into 32,000 data points with a spectral width of 20 ppm and an acquisition time of 2.7 s. Prior to the Fourier transform procedure an exponential line-broadening of 0.3 Hz was applied to the free induction decay (FID).

### Spectra Pre-processing and Statistical Analysis

^1^H-NMR spectra were analyzed by two methods: (i) a bucket method and (ii) a metabolite method.

(i) The bucket method is traditionally used to analyze ^1^H-NMR data. It consisted in converting the ^1^H-NMR spectra into a bucket intensity table with the Workflow4Metabolomics 3.3 online platform (^1^ Tremblay et al., 2014). (1) The spectra pre-processing included the following steps: solvent suppression (exclusion of the 5.1 to 4.5 ppm region corresponding to water signal), zero-filling, apodization, Fourier transform, phasing, baseline correction and calibration with TSP at 0.0 ppm. (2) The spectra alignment was performed. (3) The bucketing was defined with a 0.01 ppm interval from 0.5 to 10 ppm. The buckets corresponding to residual methanol from extraction (3.38 to 3.33 ppm) were removed. The intensity of a bucket corresponded to the intensity of the spectrum curve for that bucket. (4) The normalization was done with the whole spectrum intensity method as following:

Normalized bucket intensity = Raw bucket intensity/Whole spectrum intensity.

A table of bucket intensities was obtained with 65 rows corresponding to the animals and 758 columns corresponding to the buckets identified by their chemical shifts.

(ii) The recent metabolite method consists in converting the ^1^H-NMR spectra into a metabolite relative concentration table with the ASICS R package (R package version 4.0.2^2^) that contains an automatic approach to identify and quantify metabolites in a complex ^1^H-NMR spectrum from their unique peak pattern (fingerprint) (Tardivel et al., 2017; Lefort et al., 2019). It is a relative quantification in function of the whole spectrum and the metabolite relative concentration has no unit. The metabolite database used consisted in spectra of 176 pure metabolites described in Tardivel et al. (2017). A total of 67 metabolites were identified and quantified in at least one sample. The methanol was removed as it was used to extract the metabolites. Then only 43 metabolites were kept for further analyses as they were present in at least 50% of the samples at least at one time point. Thus the final table of metabolite relative concentration contained 65 rows corresponding to the animals and 43 columns corresponding to the metabolites.

The bucket and metabolite relative concentration tables were analyzed with SIMCA P + software (version 12, Umetrics, AB, Umea, Sweden) for carrying out multivariate statistical analysis. First, the variables were pre-processed with a Pareto normalization. A principal component analysis (PCA) was performed for finding outliers. Then partial least square analyses (PLS) were performed to explain Y variables (LW and TY) by the X variables (bucket or metabolite data). The PLS scatter plots were drawn. The latent variables that corresponded to the scores t1, t2… were new variables summarizing the X variables. The first latent variable t1 explained the largest variation of the X space. Usually the scatter plot of t1 vs t2 is a window in the X space, displaying how the X observations are situated with respect to each other. Here, as only one latent variable was created, the PLS scatter plot represented t1 on the vertical axis vs sample identification on the horizontal axis. The intensity of the Y-values were indicated by the colors of the samples. The goodness-of-fits of the models were estimated by the proportion of cumulative explained variance (*R*^2^) for both the X variables (X = buckets or metabolites) and the Y variable (Y = LW or TY) and by the predictive ability of the model (*Q*^2^). *Q*^2^ is calculated by a cross-validation. The data were divided into 7 parts and each 1/7th in turn was removed. A model was built on the 6/7th of the data until all the data have been predicted. The predictive data are then compared with the original data and the sum of squared errors calculated for the whole dataset. The Root Mean Square Error of Estimation (RMSEE) was computed and indicated the fit of the observations to the model. The Root Mean Square Error after cross validation (RMSECv) was computed. The RMSECv has to be close to the RMSEE. The plot of the Y observed vs Y predicted values were drawn for each PLS model. With a good model all the points fall close to the 45 degree line. The validation of the PLS model was evaluated by comparing the goodness of fit (*R*^2^Y and *Q*^2^) of the original model with the goodness of fit of 500 models based on data where the order of the Y-observations has been randomly permuted, while the X-matrix (bucket or metabolite) has been kept intact. The permutation plot shows, for a selected Y-variable (LW or TY), on the vertical axis the values of *R*^2^Y and *Q*^2^ for the original model and for the Y-permuted models. The horizontal axis shows the correlation between the permuted Y-vectors and the original Y-vector for the selected Y (LW or TY). The original Y has a correlation 1.0 with itself. The criteria for validity of the original model is, all permuted *Q*^2^-values are lower than the original points and the regression line of the *Q*^2^-points intersects the vertical axis at, or below zero. The latent variables associated with interesting axes were analyzed by the variable importance in projection (VIP) method. The variables (buckets or metabolites) with a VIP superior to 1 were considered as “important”. Then, a one-by-one regression with either the LW effect or the TY effect was performed on the whole datasets. The P-values were corrected for multiple tests with the Benjamini-Hochberg correction using the R software (version 3.6.1) and named “BH *P*-values”. A variable was considered as “significant” when the BH P-value was inferior to 0.050 and “tended to be significant” when the BH *P*-Value was between 0.050 and 0.100. For the buckets with VIP superior to 1, the corresponding metabolites were identified manually by importing the chemical shift lists into the Human Metabolome Database^3^ (Wishart et al., 2009). All carbohydrates identified were D-carbohydrates and all amino acids were L-amino acids. To simplify the names of the metabolites, the “D-” and the “L-” were removed before the names of the carbohydrates and the amino acids, respectively. To confirm the identification of the metabolites, the ^1^H-NMR peaks of these metabolites were manually checked on the sample spectra of some plasma samples with TopSpin software (version 4.0, Bruker BioSpin, Germany). For each metabolite all ^1^H-NMR peaks were listed. For each ^1^H-NMR peak the VIP values and the BH P-values of the corresponding buckets were summarized by the number of buckets with VIP superior to 1 and by the range of BH P-values, respectively. The relative concentrations (RC) of a metabolite with the bucket data were estimated with a method adapted from Kostidis et al., 2017, by the following formula:

Metabolite RC _*j*_ = mean [(intensity Peak _*i*__*j*_)/(H number Peak _*i*__*j*_)]

Where j represents a specific metabolite.

Where i represents each proton peak of the ^1^H-NMR spectrum of the j metabolite.

Where “intensity Peak _*i*__*j*_” was computed as the sum of the bucket intensity of the i peak for the j metabolite.

Where “H number Peak _*i*__*j*_” corresponds to the number of protons that corresponds to the i peak for the j metabolite.

Then the lists of the biomarkers obtained by the bucket method and the metabolite method were compared with a Venn diagram^4^. All the biomarkers identified by the bucket method and/or the metabolite method were considered as biomarkers. A network analysis based on the correlation of the biomarker RC and the Y-variable (LW or TY) was performed with the functions pls and network of the MixOmics R package (Lê Cao et al., 2009; Rohart et al., 2017) (R package version 4.0.2.^5^).

## Results

The rearing of male mule ducks that were overfed during 6 to 12 days enabled to obtain animals with large variability of performances. The liver of ducks weighed between 302.3 and 914.9 g and the technological yield was between 54.8 and 99.5% (Figures 1B,C). This experimental protocol mimicked the high variability of the liver characteristics that are present in the *foie gras* industry and was suitable to identify plasmatic biomarkers of *foie gras* quality by ^1^H-NMR analysis.

### 1-Identification of Plasmatic Biomarkers of Liver Weight of Foie Gras

**FIGURE 2:**
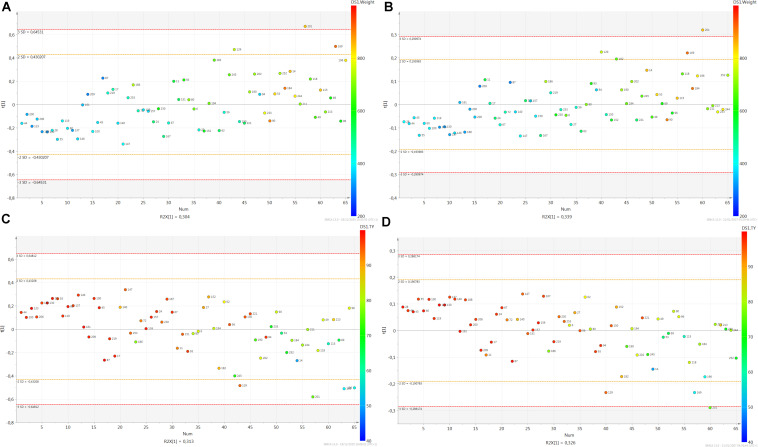
PLS score plots for foie gras **(A)** for liver weight with the bucket method (R^2^X = 0.304, R^2^Y = 0.376, Q^2^ = 0.283) and **(B)** for liver weight with the metabolite method (R^2^X = 0.339, R^2^Y = 0.323, Q^2^ = 0.269), **(C)** for technological yield with the bucket method (R^2^X = 0.313, R^2^Y = 0.298, Q^2^ = 0.175) and **(D)** for technological yield with the metabolite method (R^2^X = 0.326, R^2^Y = 0.254, Q^2^ = 0.159). The numbers correspond to the identification of the samples and the colors to the liver weight values. The legend is indicated on the right of the figures.

**FIGURE 3:**
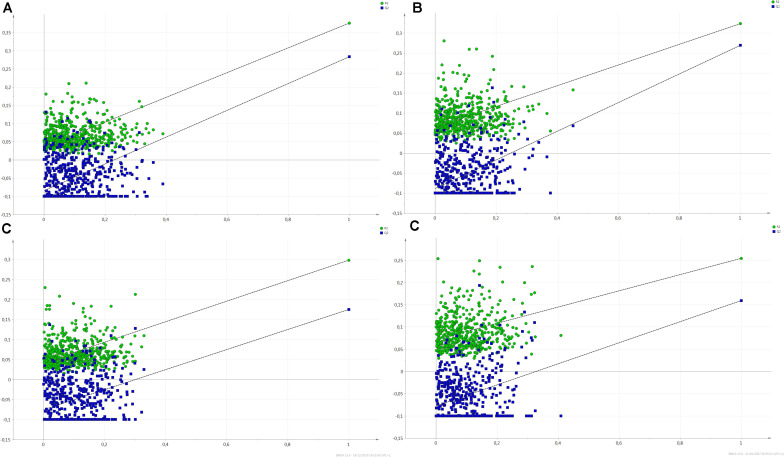
Permutation plots. **(A)** for liver weight with the bucket method, **(B)** for liver weight with the metabolite method, **(C)** for technological yield with the bucket method, **(D)** for technological yield with the metabolite method. 500 permutations were performed. The permutation plot shows, for a selected Y-variable, on the vertical axis the values of R^2^Y and Q^2^ for the original model and for the Y-permuted models. The horizontal axis shows the correlation between the permuted Y-vectors and the original Y-vector for the selected Y. The original Y has a correlation 1.0 with itself.

**FIGURE 4:**
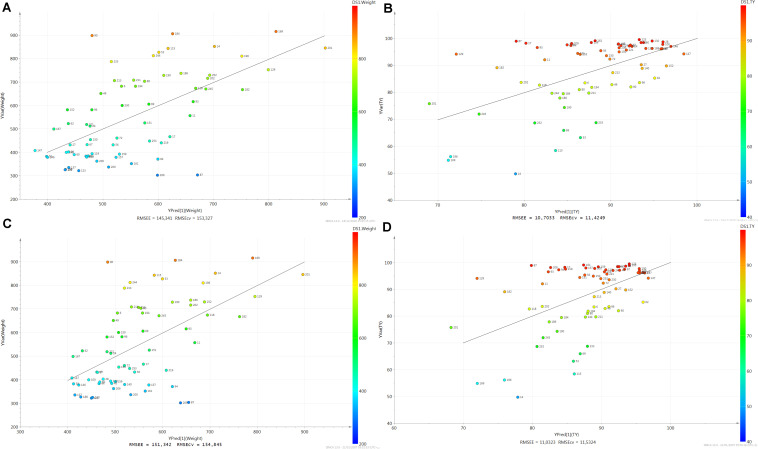
Plots of predicted vs observed data. **(A)** for liver weight with the bucket method, **(B)** for liver weight with the metabolite method, **(C)** for technological yield with the bucket method, and **(D)** for technological yield with the metabolite method. The Root Mean Square Error of Estimation (RMSEE) and the Root Mean Square Error after cross validation (RMSECv) are indicated.

First plasmatic biomarkers of LW were identified with the bucket method. After a PCA no outlier was detected (not shown). The PLS scatter plot had only one latent variable and the parameters were cumulative R^2^X = 0.304 and R^2^Y = 0.376. The projection of the samples highlighted an evolution of LW with the first latent variable on the vertical axis (Figure 2A). The prediction of the model was *Q*^2^ = 0.283. The original R^2^Y and *Q*^2^-values were higher than the ones obtained after permutation and the regression line of *Q*^2^-points intersected the vertical axis below zero (Figure 3A). The RMSEE and the RMSECv were close (145.3 and 153.3, respectively, Figure 4A). A group of 124 buckets with a VIP > 1 explained the latent variable (Supplementary Data 1). For the buckets with VIP > 1, the involved metabolites were identified. They corresponded to 14 metabolites summarized in Table 1. The relative concentrations of the metabolites were computed and the BH P-values of the metabolites were presented in bold in Table 1. In total, 13 out of the 14 metabolites were statistically significant (BH *P*-Value < 0.05) and were further considered as biomarkers of LW. For each biomarker, the numbers of important peaks that contained at least one bucket with VIP > 1 in comparison to the numbers of ^1^H-NMR peaks were indicated in parenthesis. The biomarkers of LW were 3 carbohydrates: lactate (HMDB0000190, with 2/2 important peaks), mannose (HMDB0000169 with 8/9) and sorbitol (HMDB0000247 with 5/5), 8 amino acids: alanine (HMDB0000161 with 2/2), arginine (HMDB0000517 with 4/4), glutamic acid (HMDB0000148 with 8/8), glutamine (HMDB0000641 with 3/3), isoleucine (HMDB0000172 with 6/6), lysine (HMDB0000182 with 6/12), methionine (HMDB0000696 with 3/3) and pyroglutamic acid (HMDB0000267 with 4/4) and also glycerol (HMDB0000131 with 3/3) and methylmalonic acid (HMDB00202 with 2/2; Table 1).

**TABLE 1:** List of the 13 biomarkers of foie gras liver weight identified with the bucket method.

**Metabolites**	**^1^H-NMR Peak^γ^**	**Chemical shift^δ^ (ppm)**	**BH *P*-Value^ζη^**	**Number of buckets with VIP > 1^ε^**
Carbohydrates				
**Lactate**			**0.001**	
HMDB0000190	doublet	1.31–1.32	0.001	5
	quartet	4.08–4.12	0.001 to 0.020	7
**Mannose**			**0.030**	
HMDB0000169	multiplet	3.35–3.38	0.001	1
	triplet	3.54–3.58	0.005	1
	multiplet	3.63–3.67	< 0.001	4
	multiplet	3.71–3.77	0.001 to 0.300	5
	multiplet	3.78–3.79	0.004 to 0.007	2
	multiplet	3.80–3.86	< 0.001 to 0.200	5
	multiplet	3.87–3.89	<0.001	1
	multiplet	3.91–3.94	0.001	2
	doublet	5.17		0
**Sorbitol**			**0.040**	
HMDB0000247	multiplet	3.58–3.67	< 0.001 to 0.005	9
	doublet	3.72–3.73	0.300	2
	multiplet	3.74–3.79	0.001 to 0.300	6
	doublet	3.81–3.82	< 0.001	1
	singlet	3.83	0.200	1
**Xylitol**			**0.230**	
HMDB0002917	multiplet	3.62–3.65	< 0.001	4
	multiplet	3.70–3.73	0.300	2
	multiplet	3.79–3.82	< 0.001 to 0.007	3
Amino Acids				
**Alanine**			** < 0.001**	
HMDB0000161	doublet	1.46–1.47	0.060	2
	quartet	3.75–3.79	0.001 to 0.300	4
**Arginine**			**0.002**	
HMDB0000517	multiplet	1.61–1.75	0.090	1
	multiplet	1.87–1.93	0.003 to 0.009	2
	triplet	3.22–3.25	0.005 to 0.010	3
	triplet	3.74–3.77	0.001 to 0.300	4
**Glutamic Acid**			** < 0.001**	
HMDB0000148	multiplet	2.00–2.15	<0.001 to 0.001	2
	singlet	2.29	0.003	1
	singlet	2.31	0.003	1
	doublet	2.32–2.33	< 0.001 to 0.003	2
	doublet	2.34	0.001	1
	doublet	2.35–2.36	< 0.001 to 0.002	2
	singlet	2.39	0.002	1
	quartet	3.74–3.76	0.001 to 0.300	5
**Glutamine**			** < 0.001**	
HMDB0000641	multiplet	2.09–2.16	<0.001 to 0.030	8
	multiplet	2.39–2.49	0.060 to 0.070	2
	triplet	3.75–3.78	0.001 to 0.300	6
**Isoleucine**			** < 0.001**	
HMDB0000172	triplet	0.91–0.94	0.003 to 0.006	2
	doublet	0.99–1.00	0.007 to 0.020	2
	multiplet	1.21–1.28	0.01 to 0.050	6
	multiplet	1.42–1.49	0.060	2
	multiplet	1.93–2.00	< 0.001 to 0.009	8
	doublet	3.65–3.66	<0.001	3
**Lysine**			**0.002**	
HMDB0000182	multiplet	1.38–1.52	0.001 to 0.060	4
	multiplet	1.68–1.74	0.090	1
	singlet	1.83		0
	singlet	1.85		0
	doublet	1.86–1.87		0
	doublet	1.87–1.88		0
	triplet	1.89–1.90		0
	doublet	1.91	0.009	1
	singlet	1.93	0.009	1
	singlet	1.94	0.003	2
	triplet	3.00–3.03	0.080	0
	triplet	3.73–3.75	0.003 to 0.300	4
**Methionine**			**0.030**	
HMDB0000696	multiplet	2.07–2.16	< 0.001 to 0.030	11
	triplet	2.62–2.65	0.010 to 0.020	2
	triplet	3.84–3.86	< 0.001 to 0.20	4
**Pyroglutamic Acid**			**<0.001**	
	multiplet	1.99–2.05	< 0.001 to 0.002	9
HMDB0000267	multiplet	2.35–2.43	<0.001 to 0.070	6
	multiplet	2.46–2.53	0.020 to 0.070	3
	quartet	4.16–4.19	< 0.001	1
Other organic compound				
**Glycerol**			** < 0.001**	
HMDB0000131	quartet	3.53–3.57	0.005 to 0.200	2
	quartet	3.62–3.66	< 0.001	4
	multiplet	3.75–3.79	0.003 to 0.300	6
**Methylmalonic Acid**			** < 0.001**	
HMDB00202	doublet	1.22–1.24	0.010 to 0.050	5
	quartet	3.14–3.18	0.080	1

*^γ^For each metabolite the nature of each ^1^H-NMR peak is mentioned. ^δ^For each metabolite the range of chemical shift of each peak is mentioned in ppm. ^ζ^For each bucket, the effect of the bucket intensity on the liver weight was tested by a linear model with R software and the P-Values were corrected with the Benjamini-Hochberg procedure and named BH P-Values. For each metabolite the range of BH P-Values of each peak is presented. ^η^For each biomarker the metabolite relative concentration was computed with bucket data. The effect of the metabolite relative concentration on the liver weight was tested by a linear model with R software and the P-Values were corrected with the Benjamini-Hochberg procedure and named BH P-Values. For each biomarker, the BH P-Value is indicated in bold. ^ε^The PLS model to describe the liver weight with bucket data was plotted. The first latent enabled to separate the fatty livers in function of their liver weight. The VIP of the buckets involved in the first latent were extracted. For each ^1^H-NMR peak of each metabolite, the number of buckets with VIP > 1 is indicated.*

In parallel, the metabolite method was applied and the 65 spectra were converted into a table of 43 metabolite intensities with the ASICS R package. No outlier was detected by PCA (not shown). The PLS scatter plot had only one latent variable and the parameters were cumulative R^2^X = 0.339 and R^2^Y = 0.323 (Figure 2B). The prediction of the model was Q^2^ = 0.269. The original R^2^Y and *Q*^2^-values were higher than the ones obtained after permutation and the regression line of *Q*^2^-points intersected the vertical axis below zero (Figure 3B). The RMSEE and the RMSECv were close (10.7 and 11.4, respectively, Figure 4B). The evolution of LW was well represented on the vertical axis corresponding to the first latent variable (Figure 2B). Only 9 metabolites had a VIP > 1 and 7 metabolites were significant (BH *P*-value < 0.05) and 1 metabolite tended to be significant (BH *P*-value < 0.1) (Table 2). Only these 8 metabolites were considered as biomarkers of LW, including 2 carbohydrates: glucuronic acid (VIP = 1.03, BH *P*-value = 0.006) and lactate (VIP = 4.21, BH *P*-value = 0.008) and 6 amino acids: alanine (VIP = 1.30, BH *P*-value = 0.024), glutamine (VIP = 1.10, BH *P*-value = 0.075), glycine (VIP = 1.21, BH *P*-value = 0.007), leucine (VIP = 1.12, BH *P*-value = 0.002), proline (VIP = 2.50, BH *P*-value < 0.001) and serine (VIP = 1.08, BH *P*-value = 0.033; Table 2).

**TABLE 2:** List of the 9 biomarkers of foie gras liver weight identified with the metabolite method.

**Metabolites**	**VIP^ε^**	**BH *P*-Value^ζ^**	**R^2^**
Glucose	1.90	0.151	0.04
Glucuronic Acid	1.03	0.006	0.17
Lactate	4.21	0.008	0.15
Alanine	1.30	0.024	0.11
Glutamine	1.10	0.075	0.07
Glycine	1.21	0.007	0.15
Leucine	1.12	0.002	0.22
Proline	2.50	< 0.001	0.32
Serine	1.08	0.033	0.10

*^ζ^For each biomarker the effect of their relative concentration on the liver weight was tested by a linear model with R software and the P-Values were corrected with the Benjamini-Hochberg procedure and named BH P-Values.*

**FIGURE 5:**
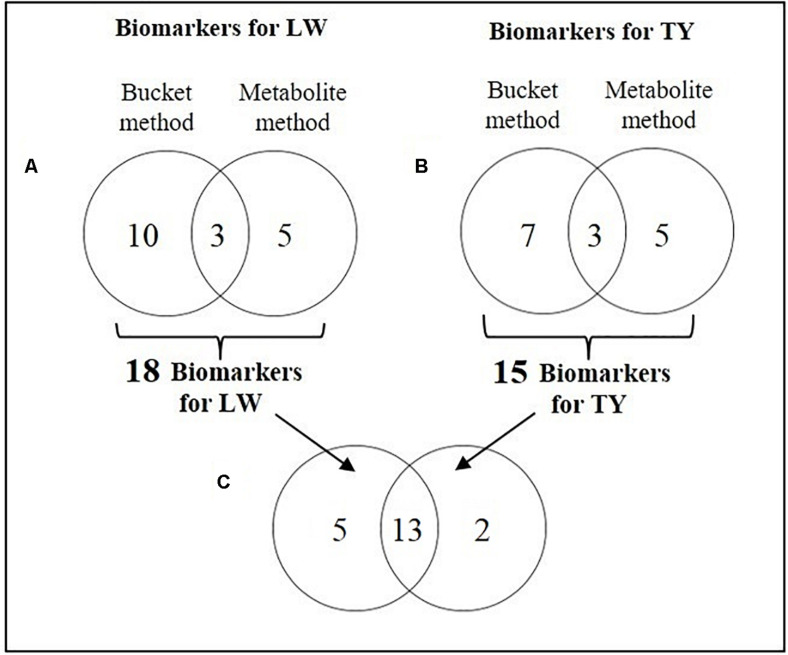
Comparisons of biomarker lists with Venn diagram. **(A)** Biomarkers of liver weight (LW) identified by the bucket method and by the metabolite method (with VIP > 1 and BH *P*-value < 0.1), **(B)**. Biomarkers of technological yield (TY) identified by the bucket method and by the metabolite method. **(C)** Biomarkers of liver weight and technological yield identified by at least one method.

**FIGURE 6:**
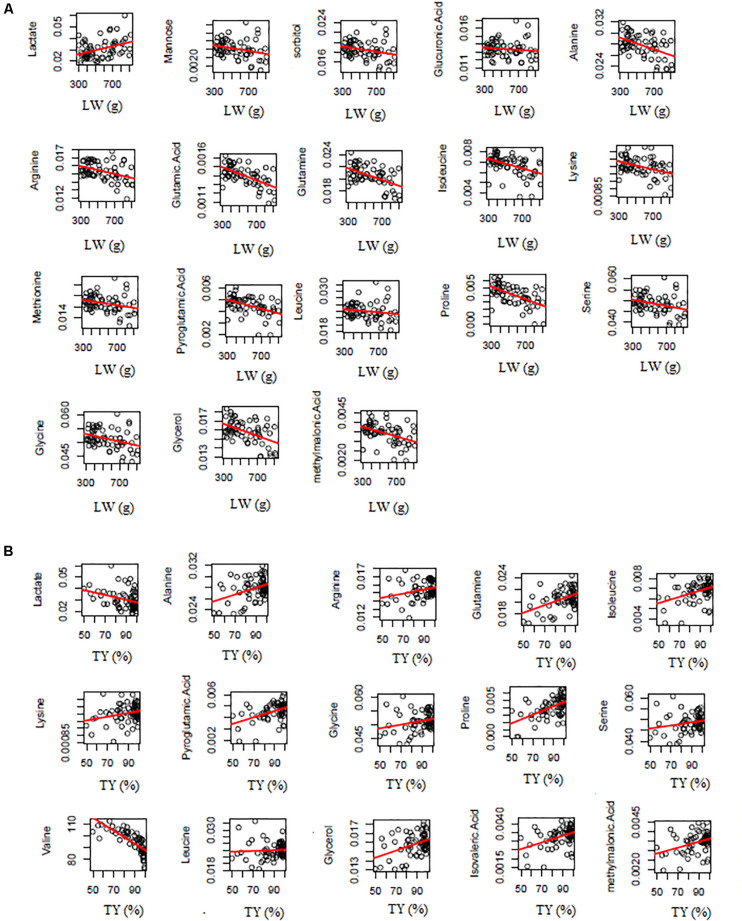
Plots of biomarker relative concentration in function of liver weight (LW) **(A)** or technological yield (TY) **(B)**. The relative concentrations are computed with the bucket data and have no unit. The regression curves are in red.

**FIGURE 7:**
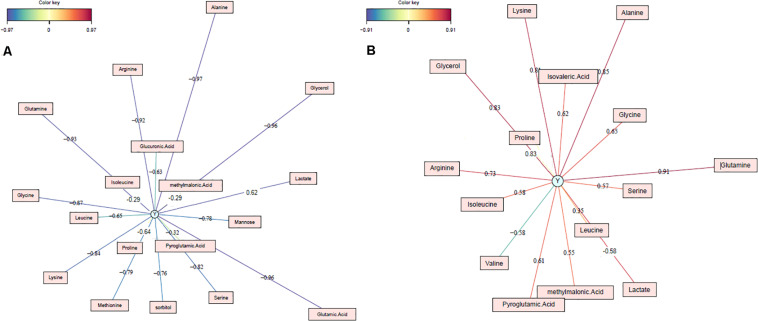
Correlation networks of foie gras biomarkers **(A)** of liver weight (Y represents the liver weight) and **(B)** of technological yield (Y represents the technological yield). The relative concentration used for the correlations are calculated with the bucket data.

In conclusion, 18 biomarkers were identified for LW whose 3 biomarkers identified by both the bucket method and the metabolite method (lactate, alanine and glutamine), 5 ones identified only by the metabolite method (glucuronic acid, glycine, leucine, proline and serine) and 10 ones identified only by the bucket method (mannose, sorbitol, arginine, glutamic acid, isoleucine, lysine, methionine, pyroglutamic acid, glycerol and methylmalonic acid; Figure 5A). For all the 18 biomarkers, their RC were computed with the bucket data and the plots of their RC in function of LW were presented in Figure 6A. The correlation network between LW and the 18 biomarkers was presented in Figure 7A. LW was positively correlated to lactate (0.62) and negatively correlated to other carbohydrates: mannose (−0.78), sorbitol (−0.76) and glucuronic acid (−0.63) and to all the amino acids: alanine (−0.97), glutamic acid (−0.96), glutamine (−0.93), arginine (−0.92), glycine (−0.87), lysine (−0.84), serine (−0.82), methionine (−0.79), leucine (−0.65), proline (−0.64), pyroglutamic acid (−0.32), isoleucine (−0.29) and to glycerol (−0.96) and methylmalonic acid (−0.29; Figure 6A).

### 2- Identification of Plasmatic Biomarkers of Technological Yield of Foie Gras

A PCA was first performed but no outlier was detected (not shown). The PLS scatter plot to explain TY had only one latent variable and the parameters were cumulative R^2^X = 0.313 and R^2^Y = 0.298. The projection of the samples highlighted an evolution of TY with the first latent variable on the vertical axis (Figure 2C). The prediction of the model was *Q*^2^ = 0.175. The original R^2^Y and *Q*^2^-values were higher than the ones obtained after permutation and the regression line of *Q*^2^-points intersected the vertical axis below zero (Figure 3C). The RMSEE and the RMSECv were close (151.3 and 154.8, respectively, Figure 4C). A group of 128 buckets with a VIP > 1 explained the latent variable (Supplementary Data 2). They corresponded to 16 metabolites (Table 3). In total, 9 out of the 16 metabolites were statistically significant (BH *P*-Value < 0.05) and 1 tended to be significant (BH *P*-value = 0.070). These 10 biomarkers were further considered as biomarkers of TY. For each biomarker, the numbers of important peaks that contained at least one bucket with VIP > 1 and the total numbers of ^1^H-NMR peaks were indicated in parenthesis. The biomarkers of TY were 1 carbohydrate: lactate (HMDB0000190 with 2/2 important peaks), 6 amino acids: alanine (HMDB0000161 with 2/2), arginine (HMDB0000517 with 4/4), glutamine (HMDB0000641 with 3/3), isoleucine (HMDB0000172 with 6/6), lysine (HMDB0000182 with 8/12) and pyroglutamic acid (HMDB0000267 with 4/4) and 3 other organic compounds: glycerol (HMDB0000131 with 3/3), isovaleric acid (HMDB0000718 with 3/3) and methylmalonic acid (HMDB00202 with 2/2; Table 3).

**TABLE 3:** List of the 15 biomarkers of foie gras technological yield identified with the bucket method.

**Metabolites**	**^1^H-NMR Peak^γ^**	**Chemical shift^δ^ (ppm)**	**BH *P*-Value^ζ^**	**Number of buckets with VIP > 1^ε^**
**Carbohydrates**				
**Lactate**			**0.001**	
HMDB0000190	doublet	1.31–1.32	0.001 to 0.060	7
	quartet	4.08–4.12	0.001 to 0.040	7
**Mannose**			**0.230**	
HMDB0000169	multiplet	3.35–3.38	0.003	1
	triplet	3.54–3.58	< 0.001 to 0.006	2
	multiplet	3.63–3.67	<0.001 to 0.004	5
	multiplet	3.71–3.77	0.010 to 0.100	3
	multiplet	3.78–3.79	0.002 to 0.020	2
	multiplet	3.80–3.86	< 0.001 to 0.003	3
	multiplet	3.87–3.89	<0.001	2
	multiplet	3.91–3.94	0.001 to 0.002	3
	doublet	5.17		0
**Sorbitol**			**0.240**	
HMDB0000247	multiplet	3.58–3.67	< 0.001 to 0.006	9
	doublet	3.72–3.73	0.030	1
	multiplet	3.74–3.79	0.002 to 0.100	5
	doublet	3.81–3.82	< 0.001 to 0.003	2
	singlet	3.83	<0.001	1
**Xylitol**			**0.690**	
HMDB0002917	multiplet	3.62–3.65	< 0.001 to 0.004	4
	multiplet	3.70–3.73	0.030	1
	multiplet	3.79–3.82	< 0.001 to 0.003	3
				
**Amino Acids**				
**Alanine**			**0.004**	
HMDB0000161	doublet	1.46–1.47	< 0.100	2
	quartet	3.75–3.79	<0.001 to 0.100	7
**Arginine**			**0.040**	
HMDB0000517	multiplet	1.61–1.75	0.060	1
	multiplet	1.87–1.93	0.010 to 0.030	2
	triplet	3.22–3.25	0.030 to 0.070	3
	triplet	3.74–3.77	0.010 to 0.100	3
**Glutamine**			** < 0.001**	
HMDB0000641	multiplet	2.09–2.16	0.001 to 0.020	7
	multiplet	2.39–2.49	0.006 to 0.030	7
	triplet	3.75–3.78	0.002 to 0.100	5
**Isoleucine**			**0.003**	
HMDB0000172	triplet	0.91–0.94	0.002 to 0.010	2
	doublet	0.99–1.00	0.004 to 0.100	3
	multiplet	1.21–1.28	0.020 to 0.100	5
	multiplet	1.42–1.49	0.100	2
	multiplet	1.93–2.00	0.001 to 0.030	7
	doublet	3.65–3.66	< 0.001 to 0.004	4
**Leucine**			**0.690**	
HMDB0000687	triplet	0.94–0.96	0.002 to 0.090	3
	multiplet	1.63–1.76	0.030 to 0.090	4
	multiplet	3.70–3.74	0.002 to 0.100	3
**Lysine**			**0.070**	
HMDB0000182	multiplet	1.38–1.52	0.006 to 0.100	3
	multiplet	1.68–1.74		4
	singlet	1.83		0
	singlet	1.85		0
	doublet	1.86–1.87		0
	doublet	1.87–1.88		0
	triplet	1.89–1.90	0.030	1
	doublet	1.91	0.030	1
	singlet	1.93	0.030	1
	singlet	1.94	0.007 to 0.010	2
	triplet	3.00–3.03	0.100	1
	triplet	3.73–3.75	0.030 to 0.100	2
**Methionine**			**0.230**	
HMDB0000696	multiplet	2.07–2.16	< 0.001 to 0.020	10
	triplet	2.62–2.65	0.050	1
	triplet	3.84–3.86	< 0.001	1
**Pyroglutamic Acid**			**0.001**	
HMDB0000267	multiplet	1.99–2.05	< 0.001 to 0.002	8
	multiplet	2.35–2.43	0.003 to 0.030	4
	multiplet	2.46–2.53	< 0.001 to 0.030	5
	quartet	4.16–4.19	<0.001	1
				
**Other organic compounds**				
**Ethanolamine**			**0.410**	
HMDB0000149	triplet	3.12–3.14	0.005 to 0.100	2
	triplet	3.80–3.82	< 0.001 to 0.003	2
**Glycerol**			**0.002**	
HMDB0000131	quartet	3.53–3.57	0.006	1
	quartet	3.62–3.66	< 0.001 to 0.004	4
	multiplet	3.75–3.79	0.002 to 0.100	6
**Isovaleric acid**			**0.001**	
HMDB0000718	doublet	0.89–0.90	0.010 to 0.050	2
	multiplet	1.90–1.98	0.002 to 0.030	6
	doublet	2.04–2.05	< 0.001 to 0.001	2
**Methylmalonic acid**			**0.005**	
HMDB00202	doublet	1.22–1.24	0.020 to 0.100	4
	quartet	3.14–3.18	0.005	1

*^γ^For each metabolite the nature of each ^1^H-NMR peak is mentioned. ^δ^For each metabolite the range of chemical shift of each peak is mentioned in ppm. ^ζ^For each bucket, the effect of the bucket intensity on the technological yield was tested by a linear model with R software and the P-Values were corrected with the Benjamini-Hochberg procedure and named BH P-Values. For each metabolite the range of BH P-Values of each peak is mentioned. ^η^For each biomarker the metabolite relative concentration was computed with the bucket data. The effect of the metabolite relative concentration on the technological yield was tested by a linear model with R software and the P-Values were corrected with the Benjamini-Hochberg procedure and named BH P-Values. For each biomarker, the BH P-Value is indicated in bold. ^ε^The PLS model to describe the liver weight with bucket data was plotted. The first latent enabled to separate the fatty livers in function of their technological yield. The VIP of the buckets involved in the first latent were extracted. For each ^1^H-NMR peak of each metabolite, the number of buckets with VIP > 1 is indicated.*

In parallel, the metabolite method was performed. No outlier was detected by PCA (not shown). The PLS scatter plot had only one latent variable and the parameters were cumulative R^2^X = 0.326 and R^2^Y = 0.254 (Figure 2D). The prediction of the model was *Q*^2^ = 0.159. The original R^2^Y and *Q*^2^-values were higher than the ones obtained after permutation and the regression line of *Q*^2^-points intersected the vertical axis below zero (Figure 3D). The RMSEE and the RMSECv were close (11.0 and 11.5, respectively, Figure 4D). The latent variable on the vertical axis explained the evolution of TY (Figure 2D) and 9 metabolites had a VIP values superior to 1 (Table 4). But only 7 metabolites were significant (BH *P*-value < 0.05) and 1 metabolite tended to be significant (BH *P*-value = 0.080). Only these 8 metabolites were considered as biomarkers of TY. There were only 1 carbohydrate: lactate (VIP = 4.03, BH *P*-value = 0.046) and 7 amino acids: alanine (VIP = 1.26, BH *P*-value = 0.080), glutamine (VIP = 1.65, BH *P*-value = 0.043), glycine (VIP = 1.39, BH *P*-value = 0.010), leucine (VIP = 1.38, BH *P*-value < 0.001), proline (VIP = 2.68, BH *P*-value < 0.001), serine (VIP = 1.33, BH *P*-value = 0.043) and L-valine (VIP = 1.13, BH *P*-value = 0.010; Table 4).

**TABLE 4:** List of the 9 biomarkers of foie gras technological yield identified with the metabolite method.

**Metabolites**	**VIP^ε^**	**BH *P*-Value^ζ^**	**R^2^**
Glucose	1.00	0.547	0.01
Lactate	4.03	0.046	0.10
Alanine	1.26	0.080	0.08
Glutamine	1.65	0.043	0.11
Glycine	1.39	0.010	0.15
Leucine	1.38	<0.001	0.25
Proline	2.68	<0.001	0.28
Serine	1.33	0.043	0.11
Valine	1.13	0.010	0.16

*^ζ^For each biomarker the effect of their relative concentration on the liver weight was tested by a linear model with R software and the P-Values were corrected with the Benjamini-Hochberg procedure and named BH P-Values.*

In conclusion, 15 biomarkers were identified for TY: 3 biomarkers were commonly identified by the bucket and the metabolite methods (lactate, alanine and glutamine), 5 ones were identified only by the metabolite method (glycine, leucine, proline, serine and valine) and 7 ones were identified by the bucket method (arginine, isoleucine, lysine, pyroglutamic acid, glycerol, isovaleric acid and methylmalonic acid; Figure 5B). For the 15 biomarkers of TY, their RC were computed with the bucket data and the plots of their RC in function of TY were presented in Figure 6B. The correlation network between TY and the biomarker RC was presented in Figure 7B. TY was negatively correlated to lactate (−0.58) and valine (−0.58) and positively correlated to glutamine (0.91), alanine (0.85), proline (0,83), lysine (0.81), arginine (0.73), glycine (0.65), pyroglutamic acid (0.61), isoleucine (0.58), serine (0.57) and leucine (0.35; Figure 6B).

Consequently, the results of the ^1^H-NMR analysis identified 18 biomarkers for the liver weight of foie gras and 15 for its technological yield (Table 5). As the phenotypic correlation between LW and TY was strong (−0.82, *P*-value < 0.001), 13 biomarkers were common to LW and TY, of which 1 carbohydrate: lactate and 10 amino acids: alanine, arginine, glutamine, glycine, isoleucine, leucine, lysine, proline, pyroglutamic acid, serine and also glycerol and methylmalonic acid (Figure 5C and Table 5). All these biomarkers were negatively correlated to LW and positively correlated to TY, except the lactate. Thus the small livers with high technological yield were characterized by a low concentration of lactate and a strong concentration of the other biomarkers and it was the contrary for the heavy livers with low technological yield. Moreover, 5 biomarkers were specific to LW: glucuronic acid, mannose, sorbitol, glutamic acid and methionine. All were negatively correlated to LW. Thus their concentrations were higher in small livers than in heavy livers. In addition, two biomarkers were specific to technological yield: the valine was negatively correlated to TY and the isovaleric acid was positively correlated to TY. As a result, the livers with high TY were characterized by a high concentration of isovaleric acid and a small concentration of valine and vice versa (Table 5).

**TABLE 5:** List of the biomarkers of liver weight and technological yield of foie gras.

	**Biomarkers of liver weight**						**Biomarkers of technological yield**					
	**With bucket method**			**With metabolite method**			**With bucket method**			**With metabolite method**		
	**Important peaks^ε^**	**BH *P*-Value^ζ^**	**Correlation with LW ^η^**	**VIP**	**BH *P*-Value**	**Correlation with LW**	**Important peaks^ε^**	**BH *P*-Value**	**Correlation with TY**	**VIP**	**BH *P*-Value**	**Correlation with TY**
**Biomarkers of LW and TY**												
Lactate	2/2	0.001	0.62	4.21	0.008	0.50	2/2	0.001	−0.58	4.03	0.046	−0.42
Alanine	2/2	<0.001	−0.97	1.30	0.024	−0.80	2/2	0.004	0.85	1.26	0.080	0.85
Arginine	4/4	0.002	−0.92				4/4	0.040	0.73			
Glutamine	3/3	<0.001	−0.93	1.10	0.075	−0.68	3/3	<0.001	0.91	1.65	0.043	0.68
Glycine			−0.87	1.21	0.007	−0.88			0.65	1.39	0.010	0.91
Isoleucine	6/6	<0.001	−0.29				6/6	0.003	0.58			
Leucine			−0.65	1.12	0.002	−0.81			0.35	1.38	< 0.001	0.86
Lysine	6/12	0.002	−0.84				8/12	0.070	0.81			
Proline			−0.64	2.50	< 0.001	−0.91			0.83	2.68	< 0.001	0.91
Pyroglutamic acid	4/4	<0.001	−0.32				4/4	0.001	0.61			
Serine			−0.82	1.08	0.033	−0.82			0.57	1.33	0.043	0.85
Glycerol	3/3	<0.001	−0.96				3/3	0.002	0.83			
Methylmalonic acid	2/2	<0.001	−0.29				2/2	0.005	0.55			
**Biomarkers of LW only**												
Glucuronic Acid			−0.63	1.03	0.006	−0.36						
Mannose	8/9	0.030	−0.78				8/9	0.230				
Sorbitol	5/5	0.040	−0.76				5/5	0.240				
GlutamicAcid	8/8	<0.001	−0.96									
Methionine	3/3	0.030	−0.79				3/3	0.230				
Biomarkers of TY only												
Valine									−0.58	1.13	0.010	0.87
Isovaleric acid							3/3	0.001	0.62			

*^ε^For each biomarker, the number of important peaks in comparison to the total number of ^1^H-NMR peaks are indicated. The important peaks contain at least one bucket with a VIP > 1 to explain the first latent variable of PLS model of liver weight or technological yield. ^ζ^For each biomarker the effect of their relative concentration computed with bucket data or metabolite data on the liver weight was tested by a linear model with R software and the P-Values were corrected with the Benjamini-Hochberg procedure and named BH P-Values. ^η^The Pearson correlation of the metabolite relative concentration obtained with bucket data or metabolite data and the liver weight or the technological yield is indicated.*

## Discussion

### NMR Methodology Discussion

A constant volume of 300 μL was sampled and prepared for further ^1^H-NMR analysis. Thus the metabolite quantity was supposed to be equivalent between all samples. To build the bucket intensity table, the intensity of each bucket was normalized by the intensity of the whole spectrum that was assumed to be equivalent in all samples, that may insert an error in the bucket intensity values. Moreover, to compute the relative concentration of a metabolite, the intensity of each ^1^H-NMR peak was divided by the number of proton for this peak as it was proposed by Kostidis et al. (2017). Then, the mean of the intensity of all ^1^H-NMR peaks for this metabolite was computed. But contrary to Kostidis et al. (2017), the concentration of a metabolite was not divided by the concentration of a standard, as the normalization of the buckets was done by the intensity of the whole spectrum, thus the bucket intensity was already a relative bucket intensity.

In addition, one of the bias of the ^1^H-NMR analysis is that several ^1^H-NMR peaks of different metabolites can have the same chemical shift. Thus there is an error in the estimation of the relative concentration of a metabolite, as the relative concentration of a peak of a metabolite can be increased by the presence of a peak of another metabolite at the same chemical shift. The fact that the relative concentration of a metabolite was computed as the mean of all the ^1^H-NMR of this metabolite may reduce this bias as all the ^1^H-NMR peaks could not be affected by the peaks of the same metabolites. Moreover, the comparison of the bucket method and metabolite method obtained with the ASICS package (Lê Cao et al., 2009; Rohart et al., 2017) enabled us to be more confident in the current results.

First O-PLS models were developed to analyze ^1^H-NMR spectra with both the bucket method and the metabolite method (not shown) but as it is known to lead to a severe overfitting of the data, we decided to use PLS models. The R^2^Y and *Q*^2^-values of our PLS models were small. Thus to validate the models, we decided to perform 500 permutation tests to compare the original R^2^Y and *Q*^2^-values to the ones obtained after permutations and we computed the RMSEE and RMSECv parameters. Moreover, the biomarkers that we identified were consistent with the literature of hepatic steatosis of other animal models, giving insights on our results on metabolic mechanisms of hepatic steatosis.

### Biomarkers Discussion

In a study to search for biomarkers of non-alcoholic fatty liver disease (NAFLD) in human, the plasmatic content of glucose was equivalent between the control and the steatosis groups but it was increased in a non-alcoholic steatosis hepatitis (NASH) group (Kalhan et al., 2011). In our study, the plasmatic glucose content was negatively correlated with LW (−0.66) but it was not significant. This difference was probably due to the fact that glucose has many ^1^H-NMR peaks and is not easy to estimate its relative concentration in plasma with ^1^H-NMR method.

The liver of mule ducks responds to overfeeding by increasing its glucose uptake capacity (Pioche et al., 2019). The glucose may be converted into sorbitol by the aldose reductase an enzyme that can reduce carbonyl function into alcohol function. The up-regulation of this enzyme induced from high glucose intake led to a strong increase of sorbitol in hepatocytes, resulting in the elevation of intracellular triglycerides (Hotta et al., 2019). In our study, the plasmatic sorbitol was negatively correlated with LW (−0.76). This result confirms the study of a rabbit model of hepatic steatosis in which a high cholesterol diet increased the presence of sorbitol and the activity of sorbitol dehydrogenase enzyme in the blood (Birkner et al., 2007). The sorbitol was already identified as a potential biomarker of alcoholic steatosis *in vivo* and *in vitro* in mice (Guo et al., 2018).

In our study, the plasmatic lactate content obtained the highest VIP values (4.21 and 4.03 for LW and TY respectively) therefore it was the metabolite with the strongest importance to draw the first latent variable that separate the livers in function of their LW or TY. It was enhanced when LW increased and reduced when TY increased (Pearson correlation of 0.62 and −0.58, respectively). Similar results were highlighted in the liver, because the liver lactate content was low in low fat loss livers that corresponded to high TY livers (Bonnefont et al., 2014). In addition, the lactate content was already identified as biomarker of liver steatosis in serum for mice (Li et al., 2011), in plasma of human (Kalhan et al., 2011) and in blood of human (Lin et al., 2020).

The amino acid metabolism was strongly impacted by the evolution of fatty livers as many amino acids were identified as biomarkers (10 of LW and TY, 2 of LW only and 1 of TY only). For all the amino acid biomarkers of LW when LW increased their plasmatic contents decreased as their correlations with LW were negative. Thus, the plasmatic amino acid concentration was higher in ducks with small livers with high TY. This corroborates with previous results as the metabolism of livers with low fat loss was oriented toward anabolism contrary to the livers with high fat loss (Theron et al., 2011; Bonnefont et al., 2014).

Many previous studies highlighted the reduction of the amino acid metabolism in animals or humans with steatosis hepatisis. For instance, in obese mice, the role of leucine on the regulation of energy metabolism was shown (Bruckbauer and Zemel, 2014; Fu et al., 2015). The role of leucine, isoleucine and valine deficiency revealed a decreasing in lipogenic gene expression in the liver in mice (Du et al., 2012). Contrary to our results, Kalhan et al. (2011) showed an increase in plasma content for glutamate and isoleucine and an insignificant evolution of leucine and valine in steatosis patients, but these evolutions were significant in NASH patients when compared to controls (Kalhan et al., 2011). Human NAFLD patients had much higher concentrations of proline in urines (Dong et al., 2017) whereas we found a higher proline concentration in plasma of ducks with low LW.

Plasmatic biomarkers of NAFLD have already been searched in many researches. For instance, Li et al. (2011) identified 3 serum biomarkers (glucose, lactate, glutamate/glutamine) to diagnose NAFLD at various stages in mice (Li et al., 2011). High plasmatic levels of isoleucine, alanine and glutamate were also associated with NAFLD severity and glutamate was the top plasmatic amino acid biomarker of obesity (Sookoian and Pirola, 2018). Glutamine supplementation reduced oxidative stress and NAFLD, and increased glucose metabolism in insulin resistant Ob/Ob mice (Leite et al., 2018). Thus the negative correlation of glutamic acid with LW (-0.96) was coherent because the heavy livers were associated to oxidative stress (Bonnefont et al., 2014). The anti-diabetic effect of pyroglutamic acid that is obtained from the glutamic acid were reported in type 2 diabetic rats and mice (Yoshinari and Igarashi, 2011). Moreover, the plasmatic contents of valine, leucine and isoleucine were significantly reduced in animals and patients with hepatic encephalopathy (Fischer et al., 1975). Our results were consistent for leucine and isoleucine (correlation with LW −0.65 and −0.29, respectively) and we did not identify valine as a biomarker of LW, but as a biomarker of TY. A lower circulating glycine levels was observed in association with hepatic insulin resistance in NAFLD patients (Alves et al., 2019) in accordance with our findings where glycine was negatively correlated with LW (−0.87) and positively correlated with TY (0.65). Moreover, the quantity of some metabolites impact directly the development of liver steatosis. Diets deficient in labile methyl groups (choline, methionine, betaine, folate) produced fatty liver (Mato et al., 2008) which validates the negative correlation of methionine with LW (−0.79), and a 5% L-Lysine diet developed fatty livers in rats (Hevia and Visek, 1980). In addition, the evolution of amino acid metabolism that we observed was consistent with the utilization by several authors of plasmatic aminotransferases as biomarkers of liver metabolic functioning (Sookoian and Pirola, 2015).

To our knowledge, there is no reference of methylmalonic acid implication in hepatic steatosis. The role of the isovaleric acid in the metabolism of hepatic steatosis is not clearly describe in the literature. However, in a study about the impact of specific starch to reverse the weight gain and hepatic steatosis induced by high fat diet in mice, the colonic isobutyric acid and isovaleric acid levels were decreased by half compared to the high-fat group of mice (Zhang et al., 2020). Even if we studied the plasma and not the colon, the high concentration of plasmatic isovaleric acid in high technological livers seemed to be consistent.

## Conclusion – Perspectives

This study presents the first analysis of plasmatic biomarkers of duck *foie gras* qualities with a large approach. We identified 18 biomarkers of liver weight and 15 biomarkers of technological yield of foie gras that were mainly lactate and amino acids. As these two quality parameters were strongly correlated, 13 metabolites were biomarkers of both LW and TY. The lactate was the most important biomarker, it increased with LW and decreased with TY. On the contrary the other biomarkers that were mainly amino acids were negatively correlated to LW and positively correlated to TY. We also identified 5 biomarkers specific to LW (3 carbohydrates and 2 amino acids) that were negatively correlated to LW. It was of main interest to identify 2 biomarkers specific to the technological yield. Contrary to the isovaleric acid, the valine was negatively correlated to TY. To predict the technological yield, these metabolites could be measured in order to optimize the valorization of *foie gras*. But further studies are required to analyze the robustness of the model based on these biomarkers.

Moreover, it was previously shown that plasmatic NEFA, TG and cholesterol were correlated to liver weight (Tavernier et al., 2017; Pioche et al., 2019). In other animal models of hepatic steatosis, lipophilic biomarkers were identified in rats (Goda et al., 2018) and specifically ceramids in mice (Dong et al., 2017) and adolescents (Maldonado-Hernández et al., 2017). Another perspective of the present study could be to search for biomarkers in the lipid fraction of the plasma.

## Data Availability Statement

The raw data supporting the conclusions of this article will be made available by the authors, without undue reservation.

## Ethics Statement

The animal study was reviewed and approved by Comité d’éthique en expérimentation animale - 073 Comité d’éthique Aquitaine Poissons Oiseaux.

## Author Contributions

ZM performed the statistical analyses and wrote the manuscript. JA made the project to be funding and supervised the animal rearing, overfeeding, and slaughtering. NM-G and CC realized the NMR analyses and the pre-processing of the data. BL helped a lot to analyze the NMR data. MM and AB helped for the analysis and the interpretation of the results. AM and CB supervised the study. All authors contributed to the article and approved the submitted version.

## Conflict of Interest

The authors declare that the research was conducted in the absence of any commercial or financial relationships that could be construed as a potential conflict of interest.

## Footnotes

1 https://workflow4metabolomics.org/

2 https://bioconductor.org/packages/ASICS/

3 http://hmdb.ca/

4 https://bioinfogp.cnb.csic.es/tools/venny/index.html

5 http://www.bioconductor.org/packages/release/bioc/html/mixOmics.html

## Supplementary Material

Supplementary Data 1 Identification of biomarkers of liver weight of duck foie gras by PLS and linear models applied on buckets of ^1^H-NMR spectra. ^ε^The PLS model to describe the liver weight with bucket data was plotted. The first latent enabled to separate the fatty livers in function of their liver weight. The VIP of the buckets involved in the first latent are presented. ^ζ^For each bucket the effect of the bucket intensity on the liver weight was tested by a linear model with R software. ^η^The *P*-Values were corrected with the Benjamini-Hochberg procedure and named BH *P*-Values.

Supplementary Data 2 Identification of biomarkers of technological yield of duck foie gras by PLS and linear models applied on buckets of ^1^H-NMR spectra. ^ε^The PLS model to describe the technological yield with bucket data was plotted. The first latent enabled to separate the fatty livers in function of their technological yield. The VIP of the buckets involved in the first latent are presented. ^ζ^For each bucket the effect of the bucket intensity on the technological yield was tested by a linear model with R software. ^η^The *P*-Values were corrected with the Benjamini-Hochberg procedure and named BH *P*-Values.
